# Assessment of the Correlation Between Two Defining Criteria for Bidirectional Isthmic Block in the Ablation of Typical Atrial Flutter

**Published:** 2011-02-07

**Authors:** R Rosu, A Abdelaal, M Andronache, G Gusetu, L Muresan, RP Martins, C Bondor, D Pop, A Malai, M Ilea, C Pop, D Dan, M Puschita, P Nanu, D Zdrenghea

**Affiliations:** 1 SCU Cluj-Napoca, Cardiology - Rehabilitation Hospital, 400347 Cluj-Napoca, Romania; 2 CHU Nancy Brabois, Department of Electrophysiology, 54500 Nancy, France; 3 UMF Cluj-Napoca, Department of Medical Data Processing and Biostatistics, 400349 Cluj-Napoca, Romania; 4 Baia Mare County Hospital, 430222, Baia Mare, Romania; 5 Arad County Hospital, 310158 Arad, Romania

**Keywords:** atrial flutter, ablation, isthmus block, electrograms

## Abstract

**Background:**

A complete, bidirectional conduction block in the cavotricuspid isthmus (CTI) represents the end-point of the typical atrial flutter ablation. We investigated the correlation between two criteria for successful ablation, one based on the atrial bipolar electrogram morphology before and after complete CTI conduction block, compared to the standard criteria of differential pacing and reversal in the right atrial depolarization sequence during coronary sinus (CS) pacing.

**Method:**

We conducted a retrospective study in 111 patients (81 males, average age 62±10 years) who underwent an atrial flutter ablation during September 2007 - July 2009 in the Cardiology - Rehabilitation Hospital, UMF Cluj-Napoca. We assessed the presence of a bidirectional block at the end of the procedure using the standard criteria. We then analyzed the morphology of the bipolar atrial electrograms adjacent to the ablation line, before and after CTI conduction block.

**Results:**

A change from a qRs morphology to a rSr' morphology when pacing from the coronary sinus and from a rsr' morphology to a QRS morphology when pacing from the low-lateral right atrium was associated with a CTI conduction block. Sensitivity (Se), specificity(Sp), positive predictive value (PPV), negative predictive value (NPV) were 96%, 89%, 99% and 67% respectively.

**Conclusion:**

Our study suggests that the analysis of the atrial bipolar electrogram next to the ablation line before and after CTI ablation may be used as a reliable criterion to validate CTI conduction block due to its high sensitivity, specificity and positive predictive value.

## Introduction

The goal of the typical atrial flutter ablation is the creation of a line of bidirectional block at the level of the cavo-tricuspid isthmus (CTI) [1].

Several criteria have been proposed for defining bidirectional block: reversal in the right atrial activation sequence during CS pacing [2], differential pacing [3], a change in the P wave morphology on surface ECG [4], the analysis of the unipolar electrogram [5], the demonstration of a large corridor of double potentials between the tricuspid valve and inferior vena cava [6], or incremental rapid pacing, as recently described by Marchlinski et al [7].

The most accurate and standard criteria is represented by the reversal in the right atrial activation sequence during CS pacing, validated by differential pacing [8], but slow conduction in the CTI can sometimes mimic bidirectional block. Therefore, more accurate criteria are needed to validate the existence of CTI block.

Lately, a criterion based on the morphology of the bipolar atrial electrograms [9] has been proposed for the evaluation of bidirectional isthmus block, but this criterion has not been sufficiently studied.

The aim of this study was to compare this new criterion with the standard criteria of bidirectional block evaluation.

## Materials and methods

A hundred and eleven patients (81 males, average age 62±10 years) who underwent radiofrequency (RF) ablation of a typical atrial flutter during September 2007 - July 2009 in the Cardiology - Rehabilitation Hospital, UMF Cluj-Napoca were included in the study.

Seventy patients had a history of ischemic cardiomyopathy, 80 patients of arterial hypertension and 8 patients of dilated cardiomyopathy. Thirty five patients had a history of atrial fibrillation. The patients in whom atrial fibrillation was induced during the ablation procedure and those in whom the instability of the catheters did not allow an accurate analysis of the intracavitary electrograms were excluded from the study.

All the procedures were performed using a previously described atrial flutter ablation technique [10]. A 20-pole mapping catheter (Livewire, St. Jude Medical) was positioned with the distal poles in the coronary sinus (CS), the rest of the poles being situated at the level of the CTI and the lateral wall of the right atrium. A bipolar catheter was positioned at the level of the His bundle. A deflectable 7F catheter with an 8 mm tip electrode, Cordis-Webster (Johnson and Johnson Inc.) or Stinger (Bard Inc.) was used as mapping and ablation catheter. The correct position of the mapping catheter was verified using a 30º left anterior oblique and 30º right anterior oblique projection. The RF energy was delivered by an Osypka RF-Generator, using 70ºC as cut-off temperature and a power of maximum 70 W.

The assessment of bidirectional CTI block was performed using the differential pacing criterion and the reversal in the right atrial depolarization sequence during CS pacing criterion.

The conduction at the level of the CTI was evaluated immediately after conversion to sinus rhythm in the cases where ablation was carried out in atrial flutter, or before the start of the ablation in the cases where the procedure was carried out in sinus rhythm. Pacing and recordings were performed in 4 points of the right atrium:

A - Lateral to the ablation line

B - Low lateral wall of the right atrium

C - The superior part of the interatrial septum

**Figure 1:**
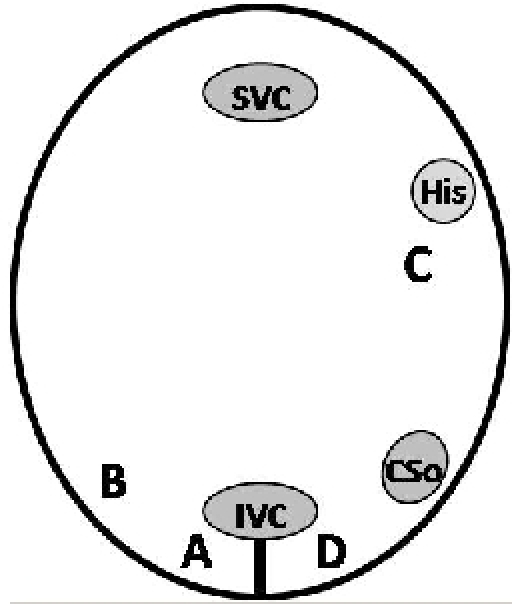
Schematic representation of pacing and recording sites in the right atrium in LAO 30º view.  SVC = Superior vena cava; His = His bundle; CSo = Coronary sinus ostium; IVC = Inferior vena cava.

D - Medial to the ablation line (Figure 1)

Bidirectional block was considered to be present when the following criteria were fulfilled:

1. Complete reversal of the right atrial depolarisation sequence when pacing from point A compared to pacing from point D.

2. AD  delay > BD delay (counter-clockwise block)

3. DA delay > CA delay (clockwise block)

4. The persistence of these criteria after a waiting period of 30 minutes.

Electrogram analysis was performed at the level of the bipole situated lateral from the ablation line when stimulation was carried out from the proximal coronary sinus, and medial from the line of block, when stimulation was carried out from the low-lateral right atrium. When a conduction block was present at the level of the CTI, a change in the polarity and morphology of the bipolar electrogram from a qRs morphology to a rSr' morphology when pacing from the coronary sinus and from a rsr' morphology to a QRS morphology when pacing from the low-lateral right atrium was analyzed near the ablation line.

### Statistical analysis

Data were analyzed using the Wilcoxon Rank test and results are displayed as average ± standard deviation. The Student t-test was used for two average comparison, and the Kolmogorov-Smirnov test was used for normal distribution evaluation. If the normal distribution hypothesis was not fulfilled, the Mann-Whitney Rank test was used. The statistical calculations were performed using SPSS 10.0.

## Results

Using the standard evaluation criteria of CTI conduction block, bidirectional block was demonstrated for 102 patients (91,9%) of the 111 patients included in the study. In 9 patients (8,1%) the block was not obtained.

For the patients in whom bidirectional block was present at the end of RF application, the activation time before and after the creation of the CTI block were: 36,9±9,2 ms and 192,4±24 ms for AD; 55,9±16 ms and 166,6±24,9 ms for BD (p<0,001); 38,8±10 ms and 193±23 ms for DA; 65,6±34,9 ms and 164,4±23 ms for CA (p<0,001). In the cases where the conduction block was not created, these intervals were: 33,7±8,2 ms and 46,2±10 ms for AD, 50,7±12,6 ms and 61,6±18,2 ms for BD; 35,98±9,2 ms and 49±11,7 ms for DA; 66,7±36,2 ms and 78,3±39,6 ms for CA.

**Figure 2:**
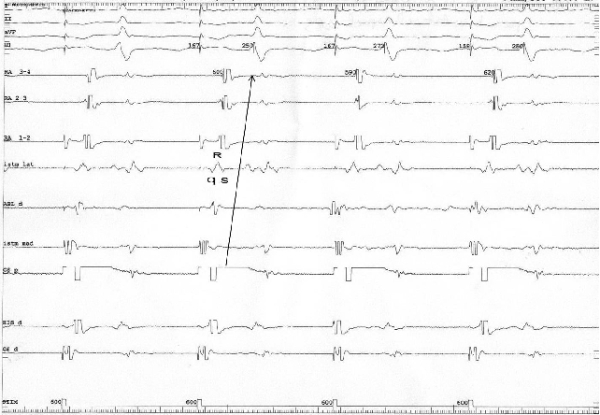
Pacing from the proximal coronary sinus before conduction block and the qRs aspect of the electrogram at the CTI level. Note the depolarization sequence before RF energy was applied, going from the proximal coronary sinus, crossing the CTI from its medial part to its lateral part and reaching the low right atrium (arrow). I, II, aVF, V1 = surface ECG leads. RA 3-4, RA 2-3, RA 1-2 = the electrograms created between the first 4 (proximal) electrodes of the duodecapolar catheter situated in the low lateral right atrium; Istm lat = the electrodes of the duodecapolar catheter situated lateral to the line of block; ABL d = the distal electrode of the ablation catheter; Istm med = the electrodes of the duodecapolar catheter situated medial to the line of block; CS p = Proximal coronary sinus; His d = Distal electrodes of the His catheter; Cs d = Distal coronary sinus.

**Figure 3:**
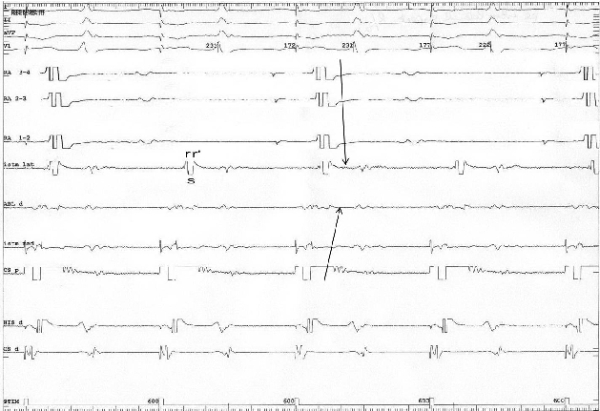
Pacing from the proximal coronary sinus after conduction block at the level of the CTI determined a reverse in the atrial depolarization sequence, with a collision of the two activation wave fronts at the level of the CTI (arrows), accompanied by a change in the atrial bipolar electrogram at the level of the lateral isthmus (Istm lat) from QRS to rSr'.  I, II, aVF, V1, RA 3-4, RA 2-3, RA 1-2, Istm lat, Abl d, Istm med, CS p , His d, CS d: same as in Figure 2.

**Figure 4:**
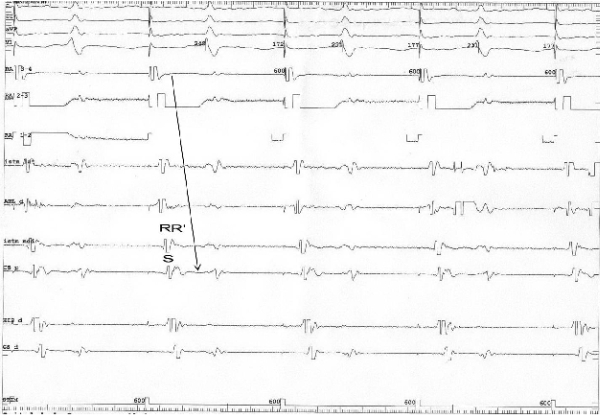
Pacing from the low lateral right atrium before conduction block and the RSR' aspect of the electrogram at the CTI level. Note the depolarization sequence before RF energy was applied, going from the low lateral right atrium, crossing the CTI from its lateral part to its medial part and reaching the proximal coronary sinus (arrow).  I, II, aVF, V1, RA 3-4, RA 2-3, RA 1-2, Istm lat, Abl d, Istm med, CS p , His d, CS d: same as in Figure 2.

**Figure 5:**
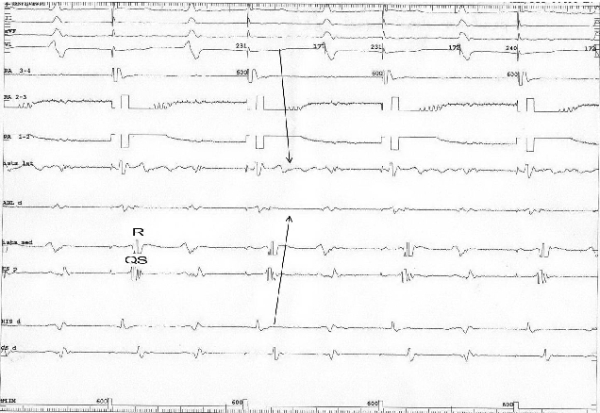
Pacing from the low right atrium after the conduction block at the level of the CTI determined a reverse in the atrial depolarization sequence, with a collision of the two activation wave fronts at the level of the CTI (arrows), accompanied by a change in the atrial bipolar electrogram at the level of the lateral isthmus (Istm med) from rSr' to QRS, II, aVF, V1, RA 3-4, RA 2-3, RA 1-2, Istm lat, Abl d, Istm med, CS p , His d, CS d: same as in Figure 2.

The studied criterion for bidirectional conduction block was analyzed in patients where block was obtained. In 98 cases (96%) there was a change form a qRs morphology to a rSr' morphology when pacing from the coronary sinus and recording from point A (Figure 2 and Figure 3), and from a rsr' morphology to a QRS morphology when pacing from point B and recording from point D (Figure 4 and Figure 5). For the rest of the 4 patients (4%) in this group, this change did not exist. Among the 9 patients in which conduction block was not present according to the standard criterion, in one case (11%) a change in the bipolar electrogram morphology was observed, from a qRs morphology to a rSr' morphology and from rsr' morphology to a QRS morphology.

**Figure 6:**
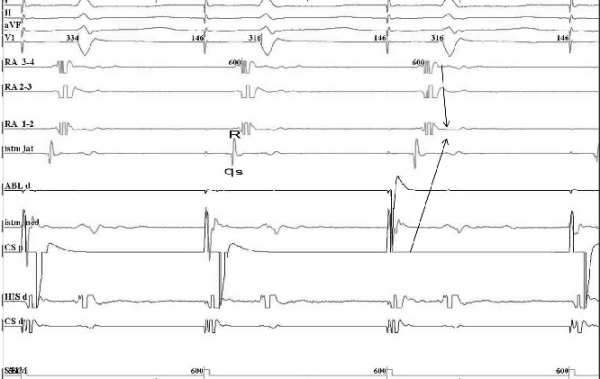
Pacing from the proximal coronary sinus demonstrating slow conduction at the CTI level. The depolarization sequence has changed since the beginning of the procedure from a collision point of the 2 propagation wavefronts at the level of the high right atrium (RA 3-4) to a collision point at the level of  low right atrium (RA 1-2) (arrows). The morphology of the bipolar atrial electrogram at the level of the lateral part of the CTI remais qRs, just like at the beginning of the procedure. I, II, aVF, V1, RA 3-4, RA 2-3, RA 1-2, Istm lat, Abl d, Istm med, CS p , His d, CS d: same as in Figure 2.

**Figure 7:**
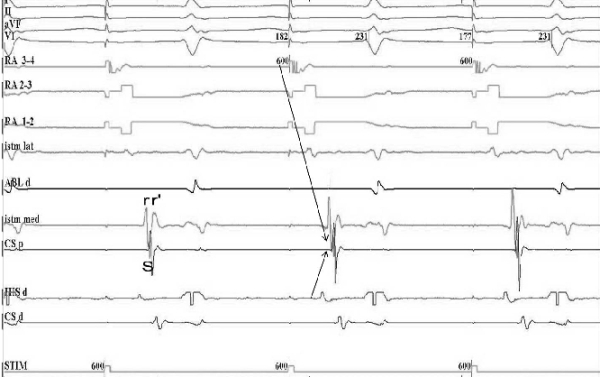
Pacing from the low right atrium showing slow conduction at the CTI level. Note the depolarization sequence, with a collision of the 2 propagation wavefronts at the level of the proximal coronary sinus (arrows). The morphology of the bipolar atrial electrogram at the level of the medial part of the CTI remais rSr', just like at the beginning of the procedure. I, II, aVF, V1, RA 3-4, RA 2-3, RA 1-2, Istm lat, Abl d, Istm med, CS p , His d, CS d: same as in Figure 2.

In the presence of slow conduction at the CTI level, before complete conduction block was achieved, we noticed a change in the atrial depolarization sequence, with a collision point at the level of low right atrium when stimulating from the proximal coronary sinus and at the level of the proximal coronary sinus when pacing from the low lateral right atrium, but with no change in the morphology of the atrial bipolar electrogram (Figure 6 and Figure 7).

The sensitivity, specificity, positive predictive value and negative predictive value of the studied criterion compared to the standard criterion, were: Se = 96,%, Sp=89%, PPV= 99% and NPV= 67%.

## Discussion

Atrial flutter is a frequent arrhythmia in clinical practice, its incidence being 180 / 100000 in general population [11]. Nowadays, the gold standard therapy is catheter ablation of the CTI [12,13].

Several criteria have been proposed for defining bidirectional block: reversal in the right atrial activation sequence during CS pacing [2], differential pacing [3], a change in the P wave morphology on surface ECG [4], the analysis of the unipolar electrogram [5], the demonstration of a large corridor of double potentials between the tricuspid valve and inferior vena cava6 and incremental rapid pacing [5].

Nevertheless, under certain circumstances, the existing criteria used for the assessment of bidirectional conduction block can lack precision in what concerns the correct evaluation of the procedural success. A complete conduction block can exist, but, in the presence of a rapid inter-caval conduction, the reversal of the right atrial activation sequence may not be evident [14-16]. In the presence of a slow conduction in the CTI, the right atrial activation sequence can be reversed, in the absence of a conduction block [17-19]. The demonstration of the existence of a corridor of large double potentials at the level of the line of block is frequently difficult to prove, due to the complex anatomy of the isthmus [20-23].

The aim of this study was to investigate the correlation between the atrial bipolar electrogram morphology before and after complete CTI conduction block and the standard criteria of differential pacing and reversal in the right atrial depolarization sequence during CS pacing.

When a conduction block was present at the level of the CTI, a change in the polarity and morphology of the bipolar electrogram from a qRs morphology to a rSr' morphology when pacing from the coronary sinus and from a rsr' morphology to a QRS morphology when pacing from the low-lateral right atrium was observed.

Andronache et al [8] also investigated the use of bipolar atrial electrogram morphology as an additional criterion identifying CTI block and noticed a clear change in the bipolar atrial electrogram polarity in the electrogram recorded by the dipoles located on the CTI and immediately lateral to the intended line of block from RS to QR pattern when pacing from the proximal coronary sinus. In our study, we did not observe a 2 component morphology on the bipolar electrogram after CTI block (QR), but a 3 component morphology (QRS or rsr'). The 3 component aspect of the bipolar atrial electrogram present in our study could be explained by the location where the atrial potential was analyzed and the direction of the depolarization front. The electrode where conduction block was evaluated was situated 2-3 mm away from the ablation line, so the depolarization sequence moved towards the analyzing electrode and away from it to the line of block. A 2 component morphology is characteristic to the final place of propagation, adjacent to the line of block, which was probably the case in Andronache's study.

Another study where the morphology of the atrial electrogram was analyzed to detect CTI block after radiofrequency ablation of the atrial flutter is the study of Villacastin at al [24]. They observed a change in the aspect of the unipolar electrogram from RS, rS, or QS to R or Rs in all patients after clockwise CTI block was obtained. However, unlike the present study, they discuss the usefulness of the unipolar electrogram to assess CTI conduction block, not the bipolar electrogram, making a comparison between their findings and ours inappropriate.

Tada et al [25] analyzed the correlation between the change in the morphology of the bipolar atrial electrograms and the presence of widely split double electrograms along the entire ablation line and found a good correlation between the two criteria.

Our results concerning the sensitivity, specificity, positive predictive value and negative predictive value are similar to results previously published in the literature [9].

In the case of the patients in which bidirectional block was present, the AD and DA intervals were higher than BD and CA intervals, as described previously by Chen et al [8]. These results were statistically significant (p<0,001).

It is possible that, in the case of the patient in which reversal of the bipolar electrogram was present but CTI conduction block was absent according to the right atrial activation sequence, only a slow conduction was obtained at the level of the isthmus and not a conduction block. Also, for the patients with proved conduction block but with no evident change in the right atrial activation sequence distal to the ablation line, this fact could have been to a non-straight ablation line.

The high sensitivity makes this criterion comparable to the standard criteria and it could be considered as a helpful method to assess procedural success. Due to its high positive predictive value, in the case of a persistent change in the bipolar electrogram morphology after the creation of a RF line at the level of the CTI, it can demonstrate the presence of the conduction block. However, the absence of this modification does not exclude the existence of a conduction block.

The main limitation of the study is the retrospective manner in which it was conducted. The present  results should be tested in a prospective study including a larger population. Also, the relationship with the recurrences in cavo-tricuspid isthmic conduction was not evaluated.

Another issue is that multiple applications of radiofrequency energy may result in a reduced amplitude of the atrial electrograms along the ablation line, which may make the evaluation of the changes in morphology difficult.

## Conclusion

Our study suggests that the changes in the atrial bipolar electrogram morphology recorded medially and laterally to the ablation line represent a powerful criterion for the evaluation of the cavo-tricuspid isthmus conduction block. This is a fast and simple method to assess the procedural success, applicable at any time of the procedure.
